# Development and Validation of a Deep Learning–based Automatic Detection Algorithm for Active Pulmonary Tuberculosis on Chest Radiographs

**DOI:** 10.1093/cid/ciy967

**Published:** 2018-11-08

**Authors:** Eui Jin Hwang, Sunggyun Park, Kwang-Nam Jin, Jung Im Kim, So Young Choi, Jong Hyuk Lee, Jin Mo Goo, Jaehong Aum, Jae-Joon Yim, Chang Min Park, Deep Learning-Based Automatic Detection Algorithm Development and Evaluation Group, Dong Hyeon Kim, Woo Woo, Choi Choi, In Pyung Hwang, Yong Sub Song, Lim Lim, Kim Kim, Jae Yeon Wi, Su Suk Oh, Mi-Jin Kang

**Affiliations:** 1 Department of Radiology, Seoul National University College of Medicine, Seoul; 2 Lunit Inc, Seoul National University Boramae Medical Center, Seoul; 3 Department of Radiology, Seoul National University Boramae Medical Center, Seoul; 4 Department of Radiology, Kyung Hee University Hospital at Gangdong, Seoul; 5 Department of Radiology, Eulji University Medical Center, Daejon; 6 Division of Pulmonary and Critical Care Medicine, Department of Internal Medicine, Seoul National University College of Medicine, Korea

**Keywords:** tuberculosis, chest radiograph, deep learning, computer-aided detection

## Abstract

**Background:**

Detection of active pulmonary tuberculosis on chest radiographs (CRs) is critical for the diagnosis and screening of tuberculosis. An automated system may help streamline the tuberculosis screening process and improve diagnostic performance.

**Methods:**

We developed a deep learning–based automatic detection (DLAD) algorithm using 54c221 normal CRs and 6768 CRs with active pulmonary tuberculosis that were labeled and annotated by 13 board-certified radiologists. The performance of DLAD was validated using 6 external multicenter, multinational datasets. To compare the performances of DLAD with physicians, an observer performance test was conducted by 15 physicians including nonradiology physicians, board-certified radiologists, and thoracic radiologists. Image-wise classification and lesion-wise localization performances were measured using area under the receiver operating characteristic (ROC) curves and area under the alternative free-response ROC curves, respectively. Sensitivities and specificities of DLAD were calculated using 2 cutoffs (high sensitivity [98%] and high specificity [98%]) obtained through in-house validation.

**Results:**

DLAD demonstrated classification performance of 0.977–1.000 and localization performance of 0.973–1.000. Sensitivities and specificities for classification were 94.3%–100% and 91.1%–100% using the high-sensitivity cutoff and 84.1%–99.0% and 99.1%–100% using the high-specificity cutoff. DLAD showed significantly higher performance in both classification (0.993 vs 0.746–0.971) and localization (0.993 vs 0.664–0.925) compared to all groups of physicians.

**Conclusions:**

Our DLAD demonstrated excellent and consistent performance in the detection of active pulmonary tuberculosis on CR, outperforming physicians, including thoracic radiologists.

## Teaser

A deep learning–based algorithm outperformed radiologists in detecting active pulmonary tuberculosis on chest radiographs and thus may play an important role in diagnosis and screening of tuberculosis in select situations, contributing to the reduction of the high burden of tuberculosis worldwide.

**(See the Editorial Commentary by Ting on pages 748–50.)**

Tuberculosis is the leading infectious cause of death worldwide; it resulted in approximately 1.7 million deaths in 2016 [1]. To reduce disease burden, the World Health Organization has recommended screening for active tuberculosis in high-risk populations [2]. In this regard, chest radiographs (CRs), which are relatively inexpensive and widely available, have played a key role in screening active tuberculosis [2], achieving a sensitivity and specificity of 98% and 75% for any abnormality and 87% and 89% for tuberculosis-related abnormalities [2–4]. Despite its promising performance, detection of tuberculosis on CRs remains a labor- and time-intensive task that requires an expert’s interpretation, which is a limited commodity in high-burden countries where medical resources and expert radiologists are scarce [5, 6]. In this context, automated detection of active pulmonary tuberculosis on CRs would be of great clinical utility.

Various approaches of automated detection have been attempted to date [5, 7–10]. In a review by Pande et al [11], a commercially available software demonstrated an area under the receiver operating characteristic curve (AUROC) of 0.71–0.84 in performance, which would be considered relatively high but suboptimal for utilization in a clinical workflow. Meanwhile, after overwhelming success in the ImageNet Large Scale Visual Recognition Competition in 2012 [12], the deep learning technique demonstrated successful results even at medical image classifications [13, 14]. As for the diagnosis of pulmonary tuberculosis on CRs, Lakhani et al. recently reported promising results in a preliminary study of 500 tuberculosis patients and 500 healthy controls in 4 datasets using the deep learning method [15]. However, their study focused on only image-wise classification of tuberculosis using a small dataset; thus, further assessments, such as localization of abnormalities, model generalizability, and performance compared to physicians, have yet to be addressed. These assessments are important in determining the practical utility of these methods, given the rapid progress in the field and the need for simple and practical improvements in tuberculosis screening and diagnosis globally.

Therefore, the purpose of our study was to develop a deep learning–based automatic detection algorithm (DLAD) for active pulmonary tuberculosis on CRs and to validate its performance using various datasets in comparison with that of physicians.

## METHODS

The institutional review boards of all participating institutions approved this study, with waiver of patients’ informed consents.

### Development of DLAD

#### Dataset

For the development of DLAD, 57c481 normal CRs from 48c986 individuals (male:female = 22c024:26c962; mean ± standard deviation age 51 ± 16 years) and 8067 CRs with active pulmonary tuberculosis (tuberculosis CRs) from 1607 patients (male:female = 908:699; mean ± standard deviation age 57 ± 17 years) were retrospectively collected from the imaging database of Seoul National University Hospital (SNUH). Normal CRs were collected via a radiology report search of CRs taken between 2010 and 2015. Tuberculosis CRs were collected from patients with newly diagnosed active pulmonary tuberculosis (via either mycobacterial culture or polymerase chain reaction [PCR] for *Mycobacterium tuberculosis*) between 2013 and 2016 who had CRs taken at time intervals of ≤1 month from the starting date of treatment. All CRs were collected regardless of the presence of corresponding chest computed tomography (CT) images in order to ensure the amount and diversity of data. All CRs were posteroanterior radiographs and obtained from various machines.

Thereafter, 3260 normal CRs and 1299 tuberculosis CRs that had been incorrectly extracted were excluded from the dataset after image labeling. A total of 54c221 normal CRs and 6768 tuberculosis CRs were used in the development of the DLAD algorithm. CR data were randomly assigned to 1 of 3 datasets: training, 53c621 normal CRs and 6468 tuberculosis CRs for optimizing network weights; tuning, 300 normal CRs and 150 tuberculosis CRs for optimizing hyperparameters; and internal validation, 300 normal and 150 tuberculosis CRs to validate in-house performance. Each dataset did not share the same patients. It should be noted that among the datasets, normal CRs had also been investigated in a previous study [16]. However, the topic of the study as well as the task and architecture of the developed algorithms were different from the present study.

#### Image Labeling and Annotation

Before the development of DLAD, all CR images were reviewed by board-certified radiologists. Normal CRs that had been read as normal in routine practice were reviewed again by 1 of 5 board-certified radiologists (7 years of experiences in reading CRs) to exclude the presence of any abnormal findings. For tuberculosis CRs, 8 board-certified radiologists (7–14 years of experiences) participated in the image labeling and annotation and ascertained whether the image findings were consistent with active pulmonary tuberculosis. Annotations for active pulmonary tuberculosis lesions were performed in 16.8% of tuberculosis CRs (1128/6768). For the training dataset, 12.8% of tuberculosis CRs (828/6468) were annotated by 2 radiologists, and all tuberculosis CRs for the tuning dataset and internal validation dataset were annotated by 5 radiologists. All annotated lesions were considered as true lesions for the training dataset, while lesions annotated by more than 3 radiologists were considered as true lesions for the tuning and internal validation datasets.

#### Development of the Algorithm

The deep convolutional neural network used in our DLAD algorithm comprised 27 layers with 12 residual connections. It was trained via a semisupervised localization approach as only a portion of the training data was annotated. The last layer of the network was split into an image-wise classification layer and a lesion-wise localization layer. The localization layer included a lung segmentation module to prevent the network from detecting lesions outside the lung. Prior to being entered into the network, CRs were randomly rescaled to cover the various lesion sizes. Image augmentation techniques such as photometric (brightness, contrast, gamma jittering, and noise injection) and geometric augmentations (horizontal flipping, cropping, and rotation) were used to make the network robust to the input from various equipment and environments. Outputs from 3 networks trained using the same data but with different hyperparameters were averaged to determine the final prediction.

Given an input CR, the classification layer of DLAD output a continuous value between 0 and 1 as the image-level probability of tuberculosis. The localization layer produced a single-channel image composed of continuous values from 0 to 1 as the per-pixel probabilities of tuberculosis overlaid on the input CR.

### Assessment of DLAD Performance

After in-house performance assessment using an internal validation dataset, external validation was performed using 6 datasets to confirm the generalization performance of DLAD. External validation datasets included retrospectively collected datasets from 4 institutions (SNUH; Boramae Medical Center; Kyunghee University Hospital at Gangdong; and Daejeon Eulji Medical Center) and 2 open-source datasets. Detailed demographic descriptions of the datasets are provided in Table 1.

**Table 1.** Demographic Description of the 6 External Validation Datasets

Demographic Variables	Seoul National University Hospital Dataset	Boramae Medical Center Dataset	Kyunghee University Hospital at Gangdong Dataset	Daejeon Eulji Medical Center Dataset	Montgomery Dataset	Shenzhen Dataset
Patients with TB						
Number of patients	83	70	103	70	52	320
Gender (male:female)	52:31	42:28	66:37	47:23	32:20	220:100
Age (years)^a^	59 (17–88)	59 (25–94)	51 (15–93)	50 (20–86)	48 (15–89)	34 (2–89)
Mode of diagnosis (culture:polymerase chain reaction only)	68:15	35:35	95:8	70:0	U/A	U/A
Time interval between diagnosis and CR (days)^a^	4 (0–14)	2 (0–13)	3 (0–30)	2 (0–31)	U/A	U/A
Time interval between CR and CT (days)^a^	7 (0–29)	1 (0–28)	3 (0–29)	0 (0–7)	U/A	U/A
Total number of TB lesions on CR	132	145	191	231	82	493
Location of TB lesion (right:left:bilateral)	36:12:35	26:8:36	34:20:49	22:10:38	17:16:19	126:69:125
Patients without TB						
Number of patients	100	70	70	100	80	326
Gender (male:female)	49:51	24:46	26:44	45:55	25:59^b^	220:106
Age (years)^a^	55 (25–80)	54 (28–86)	49.5 (15–73)	44 (19–86)	33.5 (4–70)	31 (0–85)
Time interval between CR and CT (days)^a^	0 (0–16)	0 (0–13)	4 (0–15)	0 (0–20)	U/A	U/A

Abbreviations: CR, chest radiograph; CT, computed tomography; TB, tuberculosis; U/A, unavailable.

^a^Data are median values (range).

^b^Information for 1 case was unavailable.

As for the 4 hospitals’ datasets, normal CRs and tuberculosis CRs were included. Unlike the development dataset, CRs with corresponding CT images were included in order to establish a firm reference standard for classification (ie, normal CR vs tuberculosis CR) and localization (ie, the location of tuberculosis lesion on tuberculosis CR) for precise assessment of DLAD’s performance. The inclusion criteria for tuberculosis CRs were as follows: CRs of patients diagnosed with active pulmonary tuberculosis by culture or PCR between 2016 and 2017, CRs taken with time intervals of ≤1 month from the starting date of treatment, and CRs corresponding to CT with time intervals of ≤1 month. The board-certified radiologist of each institution (7–14 years of experiences) annotated the tuberculosis lesions separately on the basis of CTs. For normal CRs, the following inclusion criteria were applied: CRs taken between May 2017 and June 2017 and CRs taken with corresponding normal CT images with time intervals of ≤1 month. Normal CTs were confirmed by the radiologists of each institution. The external validation dataset from SNUH did not share any cases with the development dataset.

Two open-source datasets were obtained from the US National Library of Medicine [17]. These datasets were from the tuberculosis screening program of Montgomery County, Maryland (Montgomery dataset), and Shenzhen, China (Shenzhen dataset), and were composed of normal CRs and tuberculosis CRs. All CRs from the 2 open-source datasets were reviewed by 2 experienced thoracic radiologists (19 and 26 years of experience) to exclude nonparenchymal tuberculosis and to annotate tuberculosis lesions. The 2 radiologists read the CRs independently initially, and the final decision was made through consensus reading in cases of discrepancy. As the DLAD targeted pulmonary tuberculosis, 6 tuberculosis CRs from the Montgomery dataset and 16 tuberculosis CRs from the Shenzhen dataset were excluded as there was only pleural effusion without evidence of pulmonary tuberculosis.

### Observer Performance Test

To compare the performances of DLAD and physicians and to determine whether DLAD can enhance physicians’ performances, an observer performance test was conducted. The reader panel comprised 15 physicians in 3 subgroups with varying degrees of experience (5 thoracic radiologists [13–26 years of experiences], 5 board-certified radiologists [5–7 years of experiences], and 5 nonradiology physicians). The SNUH dataset was used for the observer performance test. The test was performed in 2 sessions. In session 1, all readers independently assessed every CR in random order without assistance of DLAD. Physicians were asked to classify CRs as either having findings of active pulmonary tuberculosis or not and to localize the tuberculosis lesions on each CR. For localization, physicians were asked to annotate any active tuberculosis-related pulmonary abnormality, while ignoring abnormalities unlikely to be tuberculosis, and to provide their confidence levels on a 5-point scale for each annotated lesion [18]. In session 2, readers evaluated every CR again but with the assistance of DLAD. After reviewing the reader’s own initial decisions in session 1 as well as the DLAD output, each reader was asked to change or confirm their decision (including classification, localization, and confidence levels) as appropriate. (see Supplementary Figures 1 and 2 for the interface of the observer performance test.)

### Statistical Analyses

All statistical analyses were performed using R (R Project for Statistical Computing, Vienna, Austria) [19], with package RJafroc [20]. Receiver-operating characteristic (ROC) analyses were performed to evaluate image-wise classification performances, while jackknife alternative free-response ROC (JAFROC) analyses were performed to evaluate lesion-wise localization performances. For the assessment of DLAD, the image-wise probability value of each CR and maximum pixel-wise probability value in a true lesion were considered to be confidence levels for ROC and JAFROC analyses, respectively. For physicians, annotated lesions with the highest confidence level in each image were used as the confidence level for image-wise classification [21]. AUROCs and area under the alternative free-response ROC curves (AUAFROCs) were used as performance measures of ROC and JAFROC analyses, respectively. Statistical significances were evaluated using a method suggested by Dorfman et al [22]. Both the readers and cases were treated as random effects for analyses in the reader group, while only cases were treated as a random effect for analyses in individual readers [23].

In addition, sensitivities and specificities for image-wise classification as well as the true detection rate (number of correctly localized lesions/the total number of lesions) for lesion-wise localization were evaluated. To classify positive and negative tests, we defined cutoff values of the DLAD’s output probability value based on the results of our in-house validation, resulting in a high-sensitivity cutoff of 98% sensitivity for image-wise classification and a high-specificity cutoff of 98% specificity. For physicians, any detected lesion was considered positive. Comparisons of sensitivity, specificity, and true detection rates were performed using McNemar tests. Results with *P* values <.05 were considered to indicate a statistically significant difference. Holm–Bonferroni methods were used to correct multiple comparisons [24].

## RESULTS

### Assessment and Validation of DLAD Performance

In the in-house assessment using the internal validation dataset, AUROC and AUAFROC of DLAD were 0.988 (95% confidence interval [CI], 0.976–0.999) and 0.977 (95% CI, 0.966–0.988), respectively. Classification cutoff values were DLAD’s output probability values of 0.0266 for the high-sensitivity cutoff and 0.5361 for the high-specificity cutoff.

**Figure 1.**
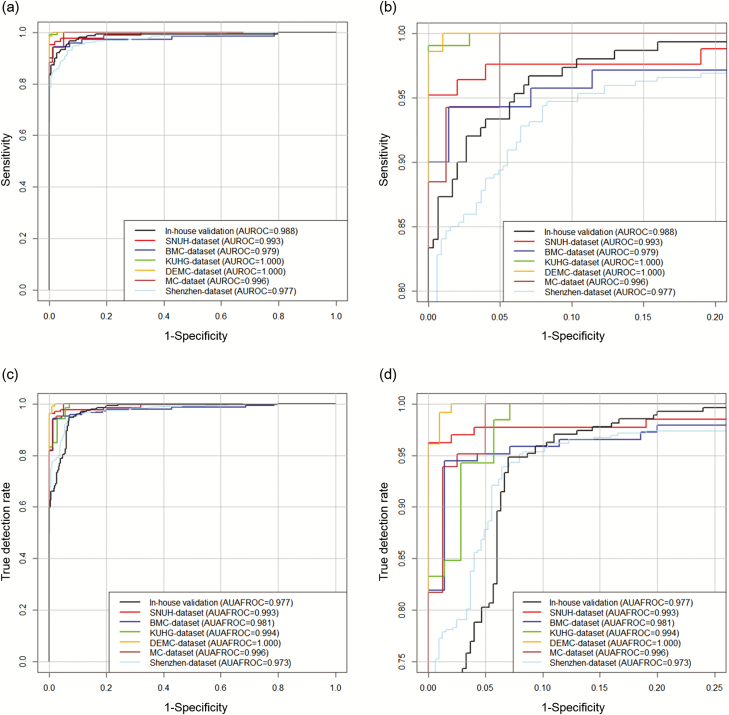
Performance of deep learning–based automatic detection algorithm (DLAD) at in-house validation and external validation. Original (a) and zoomed (b) receiver operating characteristic (ROC) curves for DLAD in in-house validation and external validation datasets. The DLAD showed consistently high performance in image-wise classification, not only in the internal validation dataset but also in the 6 external validation datasets; AUROC values ranged from 0.977 to 1.000. For lesion-wise localization performance assessed by jackknife alternative free-response ROC (c, d), DLAD showed consistently high performance in different datasets; AUAFROC ranged from 0.973 to 1.000. Abbreviations: AUAFROC, area under the alternative free-response receiver operating characteristic curves; BMC, Boramae Medical Center; DEMC, Daejeon Eulji Medical Center; KUHG, Kyunghee University Hospital at Gangdong; SNUH, Seoul National University Hospital.

Table 2 and Figure 1 show the performances of DLAD in external validation datasets. AUROCs ranged from 0.977 to1.000 and AUAFROCs ranged from 0.973 to 1.000.

**Table 2.** Performance of Deep Learning–based Automatic Detection Algorithm in the 6 External Validation Datasets

Performance Measures	Seoul National University Hospital Dataset	Boramae Medical Center Dataset	Kyunghee University Hospital at Gangdong Dataset	Daejeon Eulji Medical Center Dataset	Montgomery Dataset	Shenzhen Dataset
Area under the receiver operating characteristic curve	0.993 (0.984–1.002)	0.979 (0.954–1.005)	1.000 (0.999–1.000)	1.000 (0.999–1.000)	0.996 (0.991–1.001)	0.977 (0.967–0.987)
Area under the alternative free-response receiver operating characteristic curve	0.993 (0.983–1.003)	0.981 (0.960–1.001)	0.994 (0.987–1.001)	1.000 (0.999–1.000)	0.996 (0.990–1.002)	0.973 (0.963–0.984)
Sensitivity_SEN_^a^	0.952 (0.881–0.987)	0.943 (0.860–0.984)	1.000 (0.965–1.000)	1.000 (0.949–1.000)	1.000 (0.932–1.000)	0.947 (0.916–0.969)
Specificity_SEN_^a^	1.000 (0.964–1.000)	0.957 (0.880–0.991)	0.914 (0.823–0.968)	0.980 (0.930–0.998)	0.938 (0.860–0.979)	0.911 (0.875–0.940)
True detection rate_SEN_^a^	0.962 (0.914–0.988)	0.945 (0.894–0.976)	1.000 (0.981–1.000)	1.000 (0.984–1.000)	1.000 (0.956–1.000)	0.953 (0.931–0.970)
Sensitivity_SPE_^a^	0.843 (0.747–0.914)	0.900 (0.805–0.959)	0.990 (0.947–1.000)	0.986 (0.923–1.000)	0.846 (0.719–0.931)	0.841 (0.796–0.879)
Specificity_SPE_^a^	1.000 (0.964–1.000)	1.000 (0.949–1.000)	1.000 (0.949–1.000)	1.000 (0.964–1.000)	1.000 (0.955–1.000)	0.991 (0.973–0.998)
True detection rate_SPE_^a^	0.750 (0.667–0.821)	0.759 (0.681–0.826)	0.806 (0.743–0.860)	0.719 (0.656–0.776)	0.719 (0.609–0.813)	0.771 (0.731–0.807)

^a^Subscript SEN indicates the high-sensitivity cutoff; subscript SPE indicates the high-specificity cutoff.

### Observer Performance Test

Table 3 shows performances of physicians in sessions 1 and 2. In session 1, AUROCs for pooled nonradiology physicians, board-certified radiologists, and thoracic radiologists were 0.746, 0.946, and 0.971, respectively, while AUAFROCs were 0.664, 0.900, and 0.925, respectively. In session 2, AUROCs for 3 reader groups were 0.850, 0.961, and 0.971, respectively, while AUAFROCs were 0.781, 0.924, and 0.942, respectively.

**Table 3.** Performance of Physicians According to Reader Groups

Reader Groups	Area Under the Receiver Operating Characteristic Curve	Area Under the Alternative Free- response Receiver Operating Characteristic Curve	Sensitivity	Specificity	True Detection Rate
Session 1 (physician reading only)					
Nonradiology physicians	0.746 (0.552–0.940)	0.664 (0.466–0.861)	0.723 (0.677–0.765)	0.670 (0.627–0.711)	0.582 (0.543–0.620)
*P* value^a^	.0230	.0088			
Board-certified radiologists	0.946 (0.911–0.982)	0.900 (0.856–0.943)	0.906 (0.874–0.932)	0.948 (0.925–0.966)	0.797 (0.764–0.827)
*P* value^a^	.0082	.0003			
Thoracic radiologists	0.971 (0.948–0.993)	0.925 (0.890–0.959)	0.952 (0.927–0.970)	0.930 (0.904–0.951)	0.870 (0.842–0.894)
*P* value^a^	0.0218	0.0001			
Session 2 (physician reading with DLAD assistance)					
Nonradiology physicians	0.850 (0.694–1.005)	0.781 (0.598–0.965)	0.848 (0.810–0.881)	0.800 (0.762–0.834)	0.724 (0.688–0.758)
*P* value^b^	.0610	.0236	<.0001	<.0001	<.0001
Board-certified radiologists	0.961 (0.933–0.988)	0.924 (0.891–0.957)	0.930 (0.901–0.953)	0.954 (0.932–0.971)	0.849 (0.819–0.875)
*P* value^b^	.0606	.0353	.0075	.0833	<.0001
Thoracic radiologists	0.977 (0.957–0.997)	0.942 (0.913–0.971)	0.964 (0.941–0.980)	0.936 (0.911–0.956)	0.897 (0.871–0.919)
*P* value^b^	.1623	.0036	.0587	.2568	.0004

^a^Comparison of performance with deep learning–based automatic detection (DLAD) algorithm.

^b^Comparison of performance with session 1.

**Figure 2.**
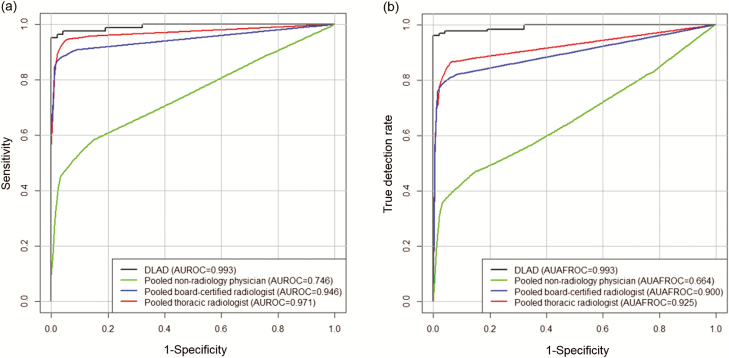
Comparison of diagnostic performance between deep learning–based automatic detection algorithm (DLAD) and physician groups. The DLAD showed significantly higher performance than all reader groups both in terms of image-wise classification (a) and lesion-wise localization (b) in the observer performance test. Abbreviations: AUAFROC, area under the alternative free-response receiver operating characteristic curves; AUROC, area under the receiver operating characteristic curve.

In the comparison between DLAD and physicians, DLAD demonstrated better performance in both AUROC and AUAFROC than all 3 reader groups (Figure 2). Compared to individual physicians, DLAD showed significantly better performance in AUROC than 13 of 15 physicians; for AUAFROC, DLAD showed significantly better performance than all of the readers.

In comparison between sessions 1 and 2, no significant improvements were observed in AUROC in any of the reader groups. However, for AUAFROC, significant improvements were observed in all 3 reader groups (see Supplementary Figure 3). Specifically, 5 readers showed significant improvements in AUROC in session 2, while 12 readers showed significant improvements in AUAFROC.

In the comparisons of the sensitivity, specificity, and true detection rates of physicians between sessions 1 and 2, pooled nonradiology physicians showed significantly improved sensitivity, specificity, and true detection rates in session 2, while pooled board-certified radiologists showed significantly improved sensitivity and true detection rates. The pooled thoracic radiologists showed significant improvement in true detection rate only in session 2.

**Figure 3.**
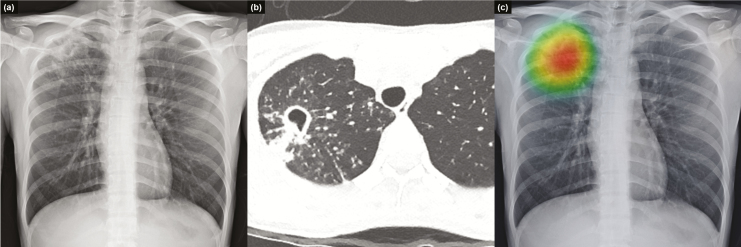
Representative case from the observer performance test. Chest radiograph of a 25-year-old woman shows a cavitary mass with multiple satellite nodules in the right upper lung field (a), which corresponded well with computed tomography images. These radiologic findings are typical for active pulmonary tuberculosis (b). Deep learning–based automatic detection algorithm provided a probability value of 0.9663 for active pulmonary tuberculosis in this case, and the classification activation map correctly localized the lesion in the right upper lung field (c).

**Figure 4.**
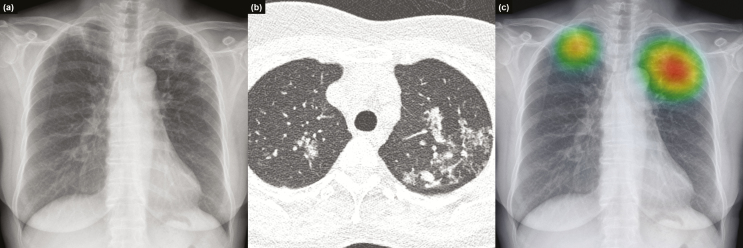
Representative case from the observer performance test. Chest radiograph of a 59-year-old female patient revealed nodular infiltrations at both lung apices (a), with a corresponding computed tomography image (b) that was initially missed by 2 readers (nonradiology physicians). Deep learning–based automatic detection algorithm (DLAD) provided a probability value of 0.9526, with a corresponding classification activation map (c). Readers who initially misclassified the chest radiograph corrected their classification after checking the results of DLAD.

**Figure 5.**
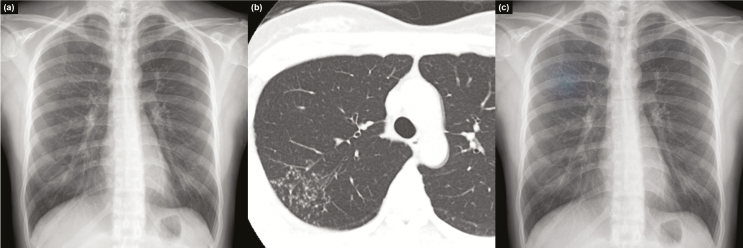
Representative case from the observer performance test. Chest radiograph of a 35-year-old female patient revealed a subtle nodular infiltration in the right upper lung field (a), with a corresponding computed tomography image (b). Deep learning–based automatic detection algorithm (DLAD) provided a probability value of 0.1625 and correctly localized the lesion (c). Seven of the 15 readers had initially missed the lesion; however, 2 readers corrected their reading after reviewing the results of DLAD.

Detailed results of individual physicians are provided in Supplementary Tables 1–3. Figures 3–5 show representative images from the observer performance test.

## DISCUSSION

We developed and validated a DLAD algorithm for active pulmonary tuberculosis on CRs that provided excellent performances not only in our in-house assessment but also in 6 independent datasets. Moreover, DLAD was demonstrated to outperform most physicians, including thoracic radiologists, both in terms of image-wise classification and lesion-wise localization. We also demonstrated improved lesion-wise localization performances of physicians with the assistance of DLAD.

The strengths of our DLAD algorithm can be summarized as follows: first, the performance of our DLAD was validated using 6 independent datasets, including CRs from different countries (Korea, United States, China) in different formats and of different qualities (Digital Imaging and Communications in Medicine data for collected datasets and Portable Network Graphics data for open-source datasets). Throughout all variations, DLAD was able to demonstrate consistently high performance (AUROC, 0.977–1.000), superior to the reported performance of a commercially available software program (CAD4TB, Image Analysis Group, Nijmegen, the Netherlands; AUROC, 0.71–0.84) [11], suggesting high generalizability of DLAD.

Second, we compared performance of DLAD with that of a diverse group of physicians, including thoracic radiologists. Although previous studies that investigated commercially available software have reported performance comparable to that of trained nonexpert human readers [25–27], its performance was reported to lag behind those of radiologists [10]. To the contrary, our study is the first to report an automated algorithm that can outperform physicians and even thoracic radiologists in the detection of active pulmonary tuberculosis.

Third, we evaluated the potential of our DLAD as a second reader, which is the most established role of computer-aided detection systems in the clinical field today [28–31], revealing that nonradiology physicians showed improvements in both sensitivity and specificity with the assistance of DLAD. Even among board-certified radiologists, DLAD was able to affect improvement in sensitivity.

Finally, our DLAD provided lesion-wise localization information in addition to image-wise classification information. Localization of each tuberculosis lesion on CR may not be as clinically relevant as image-wise classification. However, it can be important since localization can help physicians visualize the rationale behind the DLAD’s output, improving their confidence in the model. Indeed, providing an explanation for a deep learning model’s output can be essential in determining its reliability as physicians would not trust the prediction of a black box algorithm, in which the relationship between the input and output is unclear, particularly in medical applications where a single mistake can lead to substantial consequences [32]. Excellent performance in lesion-wise localization outperforming thoracic radiologists (DLAD) and improved physicians’ localization performance after reviewing the results of DLAD support that our DLAD can provide adequate visualization of the rationale behind the decision.

For clinical application of our DLAD, 2 scenarios can be considered. First, our DLAD may have the potential as a second reader in clinical practice, which would improve the performance of physicians who deal with active tuberculosis, especially in primary healthcare or community-based settings where interpretation of CRs should be done by primary care providers rather than expert radiologists. Second, the high performance of our DLAD in classifying tuberculosis CRs, outperforming even thoracic radiologists, may suggest the potential of the stand-alone utilization of DLAD in screening patients with active tuberculosis or in triaging CRs that require reading by an expert.

Our study has several limitations. First, the validation of DLAD was performed using retrospectively collected datasets that consisted of normal CRs and tuberculosis CRs. The real-world setting, however, would not be identical to that of our settings. Various abnormalities other than tuberculosis can be present, and prevalence of active pulmonary tuberculosis may be much lower than that in our test setting. Nonetheless, we believe that our results could establish a foundation for future prospective research for the verification of our DLAD in actual clinical practice. Second, the reference standards for the development datasets were defined by radiologists instead of a definitive reference such as CT. However, considering that in actual clinical practice the detection and monitoring of active pulmonary tuberculosis are performed via visual assessment of CRs by physicians, the reference standard in our investigation may accurately reflect that of a real-world situation. However, the reference standards for external validation datasets were based on CT as we believed that diagnostic performance should be measured in precisely assigned true normal CRs and tuberculosis CRs. Indeed, DLAD demonstrated excellent performances in those external validation datasets. Third, our DLAD algorithm was developed to specifically target active pulmonary tuberculosis. Thus, detection of other tuberculosis manifestation such as tuberculosis pleurisy or other significant thoracic diseases such as lung cancer was not considered. Moreover, we also do not know whether DLAD can detect radiologic abnormalities other than active pulmonary tuberculosis or whether DLAD may be able to differentiate tuberculosis from other pulmonary abnormalities. Future studies dealing with these issues are warranted.

In conclusion, our DLAD demonstrated excellent and consistent performance in the detection of active pulmonary tuberculosis on CR, outperforming most physicians, including thoracic radiologists.

## Supplementary Data

Supplementary materials are available at *Clinical Infectious Diseases* online. Consisting of data provided by the authors to benefit the reader, the posted materials are not copyedited and are the sole responsibility of the authors, so questions or comments should be addressed to the corresponding author.

ciy967_suppl_Supplementary_Figure1

ciy967_suppl_Supplementary_Figure2

ciy967_suppl_Supplementary_Figure3

ciy967_suppl_Supplementary_Material

## Notes

***Acknowledgments.*** The Deep Learning-Based Automatic Detection Algorithm Development and Evaluation Group includes the following authors: Dong Hyeon Kim, Department of Radiology, Seoul National University Hospital, College of Medicine, Seoul, Republic of Korea; Sungmin Woo, Department of Radiology, Armed Forces Daejon Hospital, Daejon, Republic of Korea; Wonseok Choi, Department of Radiology, Seoul National University Hospital, College of Medicine, Seoul, Republic of Korea; In Pyung Hwang, Department of Radiology, Seoul National University Hospital, College of Medicine, Seoul, Republic of Korea; Yong Sub Song, Department of Radiology, Seoul National University Hospital, College of Medicine, Seoul, Republic of Korea; Jiyeon Lim, Department of Radiology, Seoul National University Hospital, College of Medicine, Seoul, Republic of Korea; Hyungjin Kim, Department of Radiology, Seoul National University Hospital, College of Medicine, Seoul, Republic of Korea; Jae Yeon Wi; Department of Radiology, Asan Medical Center, Seoul, Republic of Korea; Su Suk Oh, Department of Radiology, Seoul National University Hospital, Seoul, Republic of Korea; and Mi-Jin Kang, Department of Radiology, Inje University Sanggyepaik Hospital, Seoul, Republic of Korea. We also thank Chris Woo, Bachelor of Arts, for this editorial assistance.

***Financial support.*** The work was supported by the Seoul National University Hospital Research fund (grant 04-2016-3000), Lunit Inc., and the Seoul Research & Business Development Program (grant FI170002).

***Potential conflicts of interests.*** C. M. P. reports grants from Seoul National University Hospital, Lunit Inc., and the Seoul Metropolitan Government during the conduct of the study. S. P. and J. A. are employees of Lunit Inc. All remaining authors: No reported conflicts. All authors have submitted the ICMJE Form for Disclosure of Potential Conflicts of Interest. Conflicts that the editors consider relevant to the content of the manuscript have been disclosed.
