# Supplementary figures and images for: Development and Validation of a Deep Learning–based Automatic Detection Algorithm for Active Pulmonary Tuberculosis on Chest Radiographs
Source: Clin Infect Dis. 2018 Nov 8;69(5):739–47. doi: 10.1093/cid/ciy967 (PMC6695514; doi:10.1093/cid/ciy967)

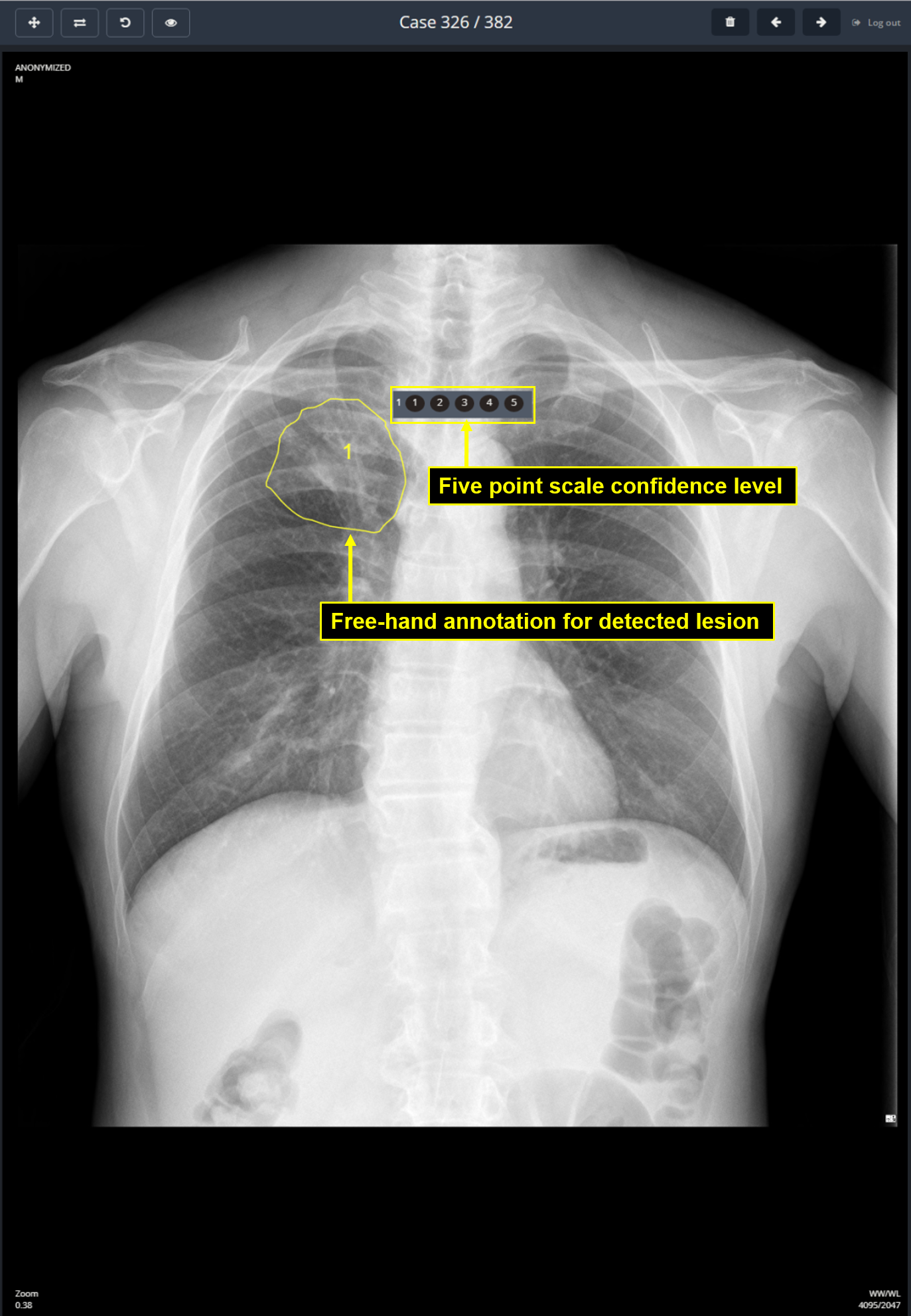

Supplement: ciy967_suppl_Supplementary_Figure1 [file ciy967_suppl_supplementary_figure1.png]

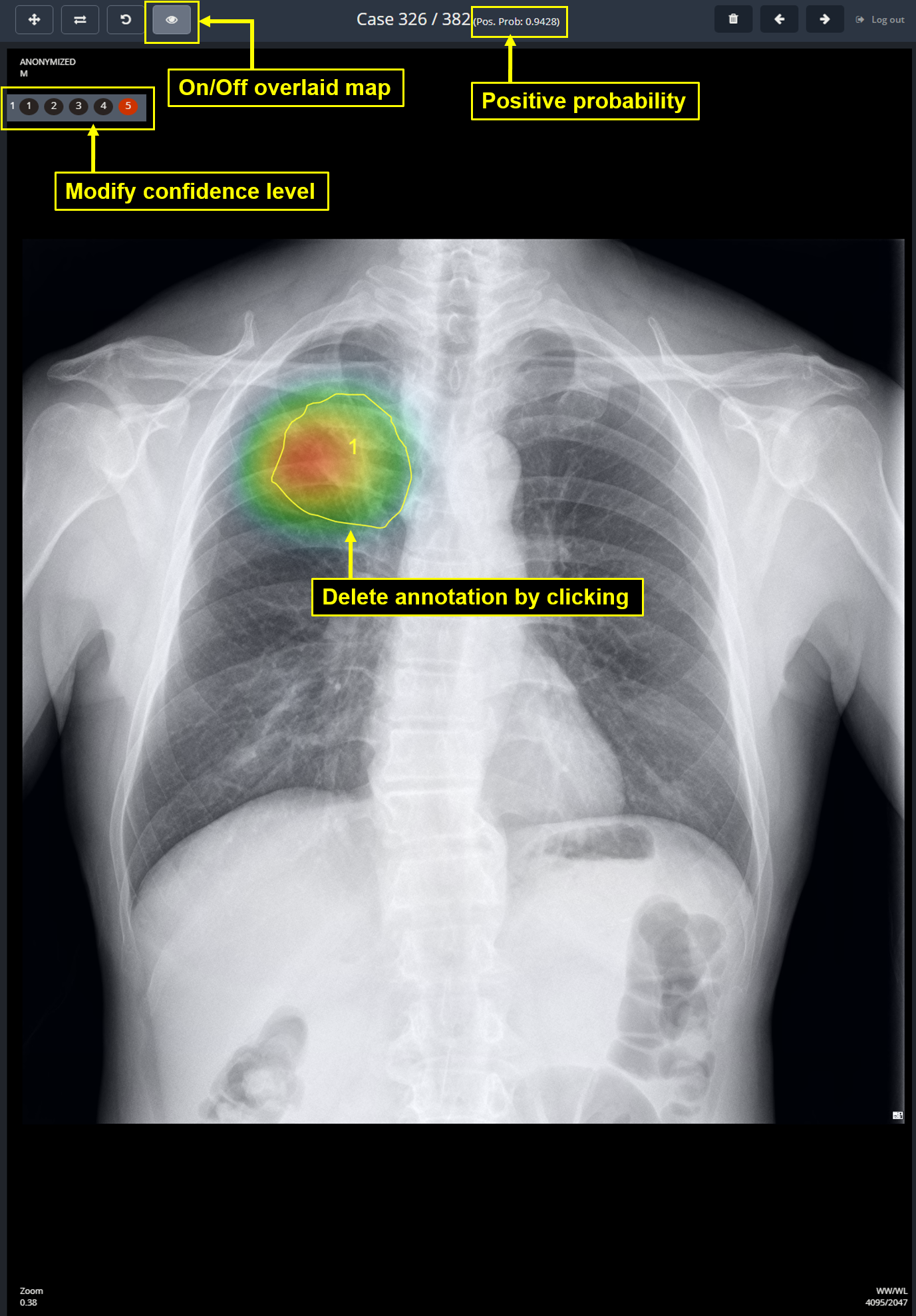

Supplement: ciy967_suppl_Supplementary_Figure2 [file ciy967_suppl_supplementary_figure2.png]

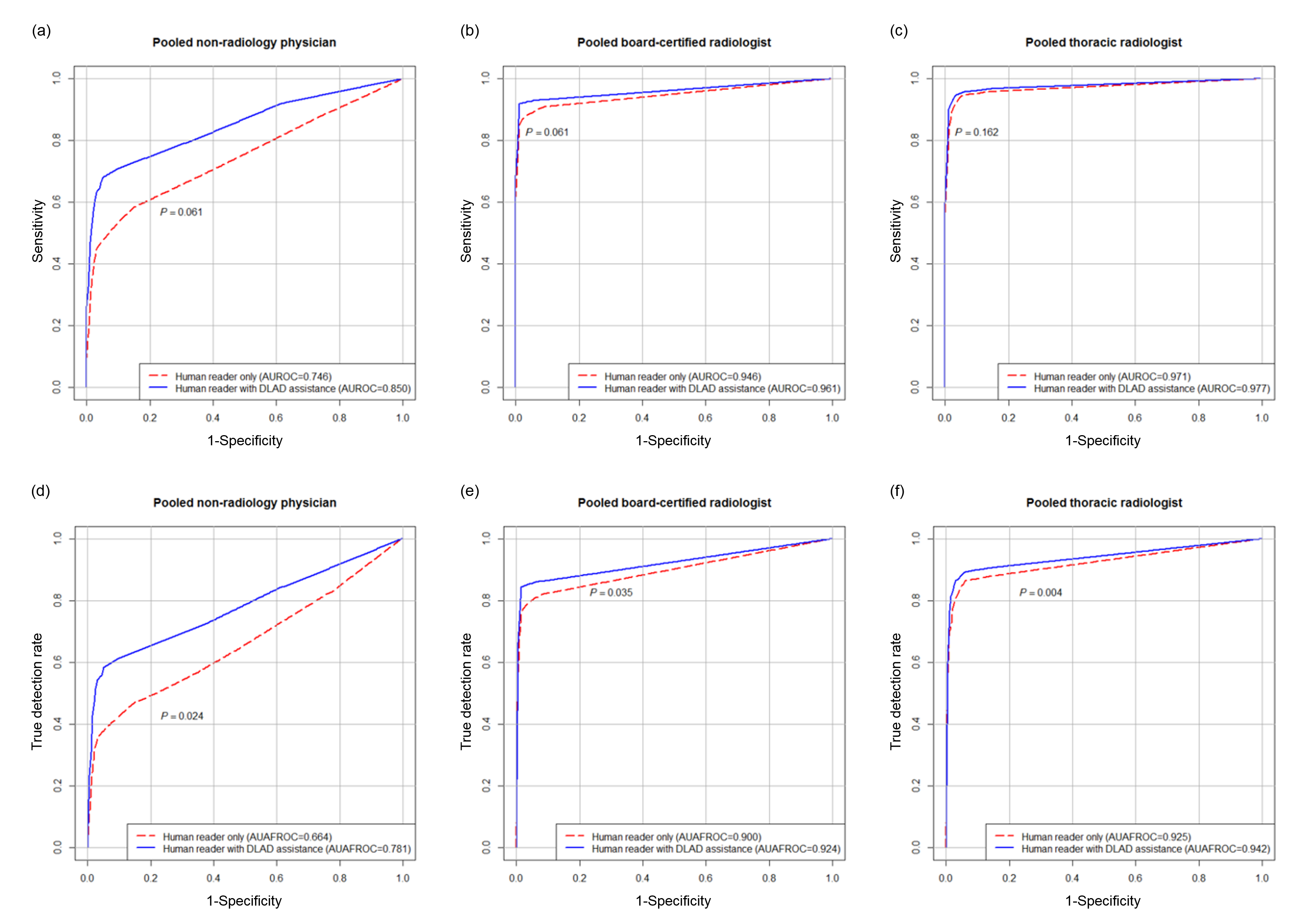

Supplement: ciy967_suppl_Supplementary_Figure3 [file ciy967_suppl_supplementary_figure3.png]
