# Supplementary material for: Development and Validation of a Deep Learning–based Automatic Detection Algorithm for Active Pulmonary Tuberculosis on Chest Radiographs
Source: Clin Infect Dis. 2018 Nov 8;69(5):739–47. doi: 10.1093/cid/ciy967 (PMC6695514; doi:10.1093/cid/ciy967)
Supplement: ciy967_suppl_Supplementary_Material [file ciy967_suppl_supplementary_material.docx]

**Supplementary material**

The online-only supplementary information includes 3 supplementary figures and 3 supplementary tables.

**Supplementary Figure 1. User interface of the observer performance test (Session 1)**

A web-based user interface was used for the observer performance test. In session 1 (physician only reading), CRs were presented without any clinical information. Window adjustment, magnification, panning, and inversion were allowed. Thereafter, any detected active pulmonary TB-related abnormalities were annotated via free-hand drawing of a closed-curve around the lesion. After the annotation, physicians recorded their confidence level for each lesion on a five-point scale.

**
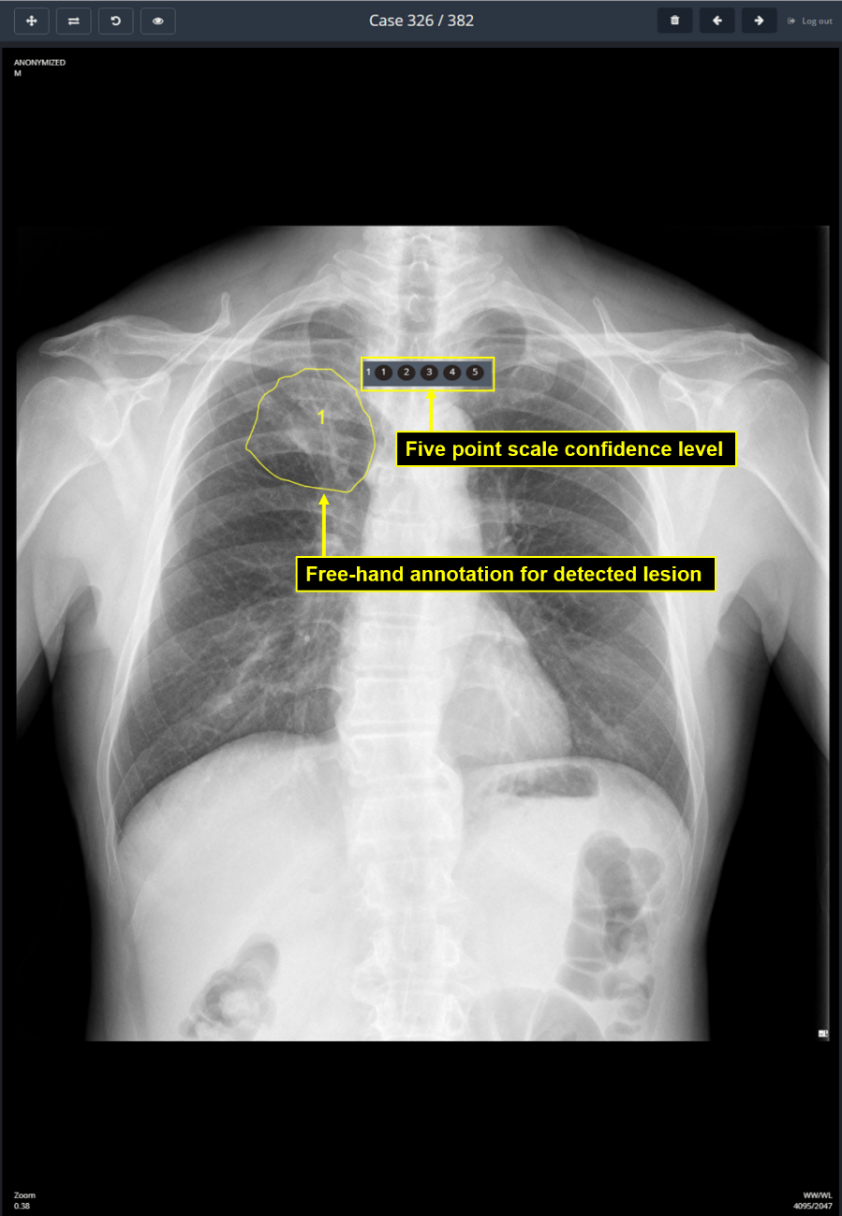
**

**Supplementary Figure 2. User interface of the observer performance test (Session 2)**

In session 2 (Physician reading with DLAD assistance), the physicians were instructed to check the positive probability of each CR as well as the DLAD-provided classification activation map overlaid on the CR image. After checking the results of DLAD, the physicians were allowed to add or remove the annotation and modify their initial confidence level from session 1, if necessary. The overlaid classification activation map could also be turned on and off.

**
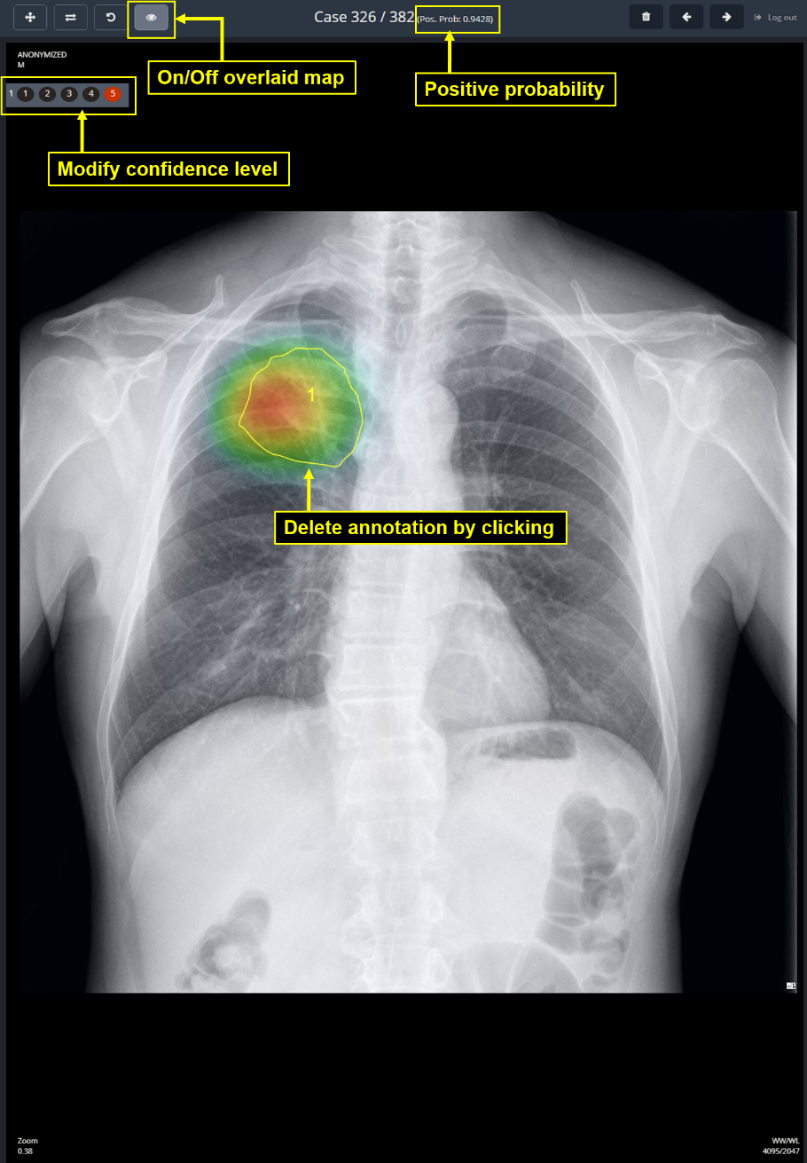
**

**Supplementary Figure 3. Comparison of diagnostic performance between physicians only and physicians with DLAD assistance**

There were no statistically significant improvements in image-wise classification performance (AUROC) with the assistance of DLAD in any of the three reader groups (a, non-radiology physicians; b, board-certified radiologists; and c, thoracic radiologists). However, for lesion-wise localization (AUAFROC), a statistically significant improvement was observed in all three reader groups (d, non-radiology physicians; e, board-certified radiologists; and f, thoracic radiologists) with DLAD assistance.


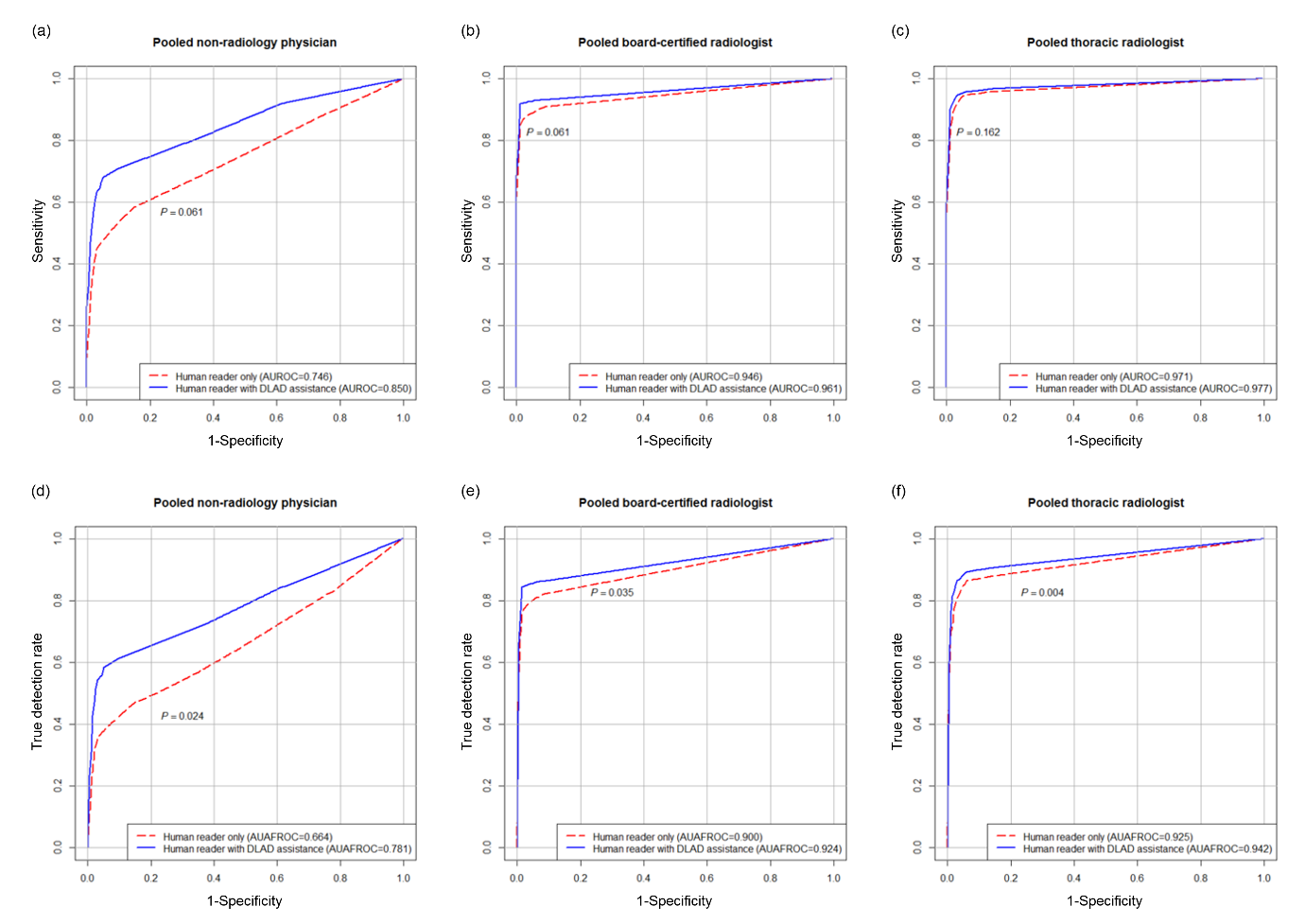


**Supplementary Table 1. Performance of individual non-radiology physician readers**

|  | AUROC | AUAFROC | Sensitivity | Specificity | True detection rate |
| --- | --- | --- | --- | --- | --- |
| Session 1 (Physician only) | | | | | |
| Non-radiology physician 1 | 0.818  (0.756-0.879) | 0.715  (0.654-0.776) | 0.771  (0.666-0.856) | 0.710  (0.611-0.796) | 0.614  (0.525-0.697) |
| *P*-value^*^ | <0.0001 | <0.0001 |  |  |  |
| Non-radiology physician 2 | 0.869  (0.820-0.919) | 0.795  (0.747-0.843) | 0.759  (0.653-0.846) | 0.960  (0.901-0.989) | 0.614  (0.525-0.697) |
| *P*-value^*^ | <0.0001 | <0.0001 |  |  |  |
| Non-radiology physician 3 | 0.534  (0.455-0.614) | 0.432  (0.361-0.503) | 0.807  (0.706-0.886) | 0.230  (0.152-0.325) | 0.621  (0.533-0.704) |
| *P*-value^*^ | <0.0001 | <0.0001 |  |  |  |
| Non-radiology physician 4 | 0.888  (0.835-0.940) | 0.809  (0.755-0.864) | 0.868  (0.775-0.932) | 0.650  (0.548-0.743) | 0.750  (0.667-0.821) |
| *P*-value^*^ | <0.0001 | <0.0001 |  |  |  |
| Non-radiology physician 5 | 0.621  (0.554-0.689) | 0.567  (0.508-0.626) | 0.410  (0.303-0.523) | 0.800  (0.708-0.873) | 0.311  (0.233-0.397) |
| *P*-value^*^ | <0.0001 | <0.0001 |  |  |  |
| Session 2 (Physician with DLAD assistance) | | | | | |
| Non-radiology physician 1 | 0.929  (0.891-0.967) | 0.888  (0.847-0.929) | 0.868  (0.775-0.932) | 0.980  (0.930-0.998) | 0.788  (0.708-0.854) |
| *P*-value^†^ | 0.0001 | <0.001 | 0.0325 | <0.0001 | <0.0001 |
| Non-radiology physician 2 | 0.928  (0.889-0.967) | 0.873  (0.830-0.916) | 0.855  (0.675-0.828) | 0.990  (0.946-1.000) | 0.758  (0.675-0.828) |
| *P*-value^†^ | 0.0031 | <0.001 | 0.0209 | 0.0833 | 0.0001 |
| Non-radiology physician 3 | 0.626  (0.549-0.703) | 0.524  (0.451-0.596) | 0.868  (0.775-0.932) | 0.410  (0.313-0.513) | 0.682  (0.595-0.760) |
| *P*-value^†^ | <0.001 | <0.001 | 0.0253 | <0.0001 | 0.0047 |
| Non-radiology physician 4 | 0.895  (0.845-0.946) | 0.840  (0.786-0.894) | 0.880  (0.790-0.941) | 0.670  (0.569-0.761) | 0.796  (0.717-0.861) |
| *P*-value^†^ | 0.1933 | 0.0058 | 0.5637 | 0.1573 | 0.1430 |
| Non-radiology physician 5 | 0.870  (0.821-0.919) | 0.781  (0.738-0.825) | 0.771  (0.666-0.856) | 0.950  (0.887-0.984) | 0.599  (0.510-0.683) |
| *P*-value^†^ | <0.0001 | <0.0001 | <0.0001 | 0.0001 | <0.0001 |

Abbreviations: AUROC, area under the receiver operating characteristic curve; AUAFROC, area under the alternative free-response receiver operating characteristic curve; DLAD, deep-learning based automatic detection algorithm.

^*^Comparison of performance with DLAD

^†^Comparison of performance with session 1

**Supplementary Table 2. Performance of individual board-certified radiologist readers**

|  | AUROC | AUAFROC | Sensitivity_1_^*^ | Specificity_1_^*^ | True detection rate_1_^*^ |
| --- | --- | --- | --- | --- | --- |
| Session 1 (Physician only) | | | | | |
| Board-certified radiologist 1 | 0.963  (0.934-0.992) | 0.920  (0.887-0.954) | 0.940  (0.865-0.980) | 0.900  (0.824-0.951) | 0.864  (0.793-0.917) |
| *P*-value^†^ | 0.0137 | <0.0001 |  |  |  |
| Board-certified radiologist 2 | 0.942  (0.906-0.977) | 0.880  (0.838-0.921) | 0.892  (0.804-0.949) | 0.960  (0.901-0.989) | 0.773  (0.692-0.841) |
| *P*-value^†^ | 0.0018 | <0.0001 |  |  |  |
| Board-certified radiologist 3 | 0.906  (0.862-0.949) | 0.854  (0.810-0.899) | 0.831  (0.733-0.941) | 0.970  (0.915-0.994) | 0.720  (0.635-0.794) |
| *P*-value^†^ | <0.0001 | <0.0001 |  |  |  |
| Board-certified radiologist 4 | 0.964  (0.936-0.993) | 0.909  (0.870-0.948) | 0.952  (0.881-0.987) | 0.920  (0.848-0.965) | 0.849  (0.776-0.905) |
| *P*-value^†^ | 0.0381 | <0.0001 |  |  |  |
| Board-certified radiologist 5 | 0.957  (0.927-0.988) | 0.935  (0.899-0.970) | 0.916  (0.834-0.965) | 0.990  (0.946-1.000) | 0.871  (0.802-0.923) |
| *P*-value^†^ | 0.0091 | 0.0005 |  |  |  |
| Session 2 (Physician with DLAD assistance) | | | | | |
| Board-certified radiologist 1 | 0.966  (0.938-0.995) | 0.936  (0.904-0.969) | 0.940  (0.865-0.980) | 0.900  (0.824-0.951) | 0.886  (0.820-0.935) |
| *P*-value^‡^ | 0.0778 | 0.0334 | 1.0000 | 1.0000 | 0.0027 |
| Board-certified radiologist 2 | 0.962  (0.933-0.991) | 0.917  (0.881-0.952) | .940  (.865-.980) | 0.960  (0.901-0.989) | 0.841  (0.767-0.899) |
| *P*-value^‡^ | 0.0590 | 0.0010 | 0.0455 | 1.0000 | 0.0027 |
| Board-certified radiologist 3 | 0.937  (0.900-0.974) | 0.901  (0.862-0.940) | 0.880  (0.790-0.941) | 0.970  (0.915-0.994) | 0.811  (0.733-0.874) |
| *P*-value^‡^ | 0.0215 | 0.0015 | 0.1024 | 1.0000 | 0.0005 |
| Board-certified radiologist 4 | 0.968  (0.940-0.995) | 0.929  (0.890-0.967) | 0.952  (0.881-0.987) | 0.940  (0.874-0.978) | 0.879  (0.811-0.929) |
| *P*-value^‡^ | 0.7174 | 0.0441 | 1.0000 | 0.1573 | 0.1025 |
| Board-certified radiologist 5 | 0.970  (0.944-0.996) | 0.936  (0.901-0.971) | 0.940  (0.865-0.980) | 1.000  (0.964-1.000) | 0.871  (0.802-0.923) |
| *P*-value^‡^ | 0.1428 | 0.8910 | 0.1573 | 0.3173 | 1.0000 |

Abbreviations: AUROC, area under the receiver operating characteristic curve; AUAFROC, area under the alternative free-response receiver operating characteristic curve; DLAD, deep-learning based automatic detection algorithm.

^*^Subscripts indicate the confidence level threshold for classification of positive prediction.

^†^Comparison of performance with DLAD

^‡^Comparison of performance with session 1

**Supplementary Table 3. Performance of individual thoracic radiologist readers**

|  | AUROC | AUAFROC | Sensitivity | Specificity | True detection rate |
| --- | --- | --- | --- | --- | --- |
| Session 1 (Physician only) | | | | | |
| Thoracic radiologist 1 | 0.980  (0.958-1.001) | 0.950  (0.917-0.983) | 0.964  (0.898-0.993) | 0.940  (0.874-0.978) | 0.909  (0.847-0.952) |
| *P*-value^*^ | 0.0782 | 0.0042 |  |  |  |
| Thoracic radiologist 2 | 0.987  (0.969-1.004) | 0.939  (0.911-0.967) | 0.976  (0.916-0.997) | 0.960  (0.901-0.989) | 0.886  (0.820-0.935) |
| *P*-value^*^ | 0.4181 | 0.0004 |  |  |  |
| Thoracic radiologist 3 | 0.969  (0.944-0.995) | 0.921  (0.883-0.960) | 0.952  (0.881-0.987) | 0.870  (0.788-0.929) | 0.879  (0.811-0.929) |
| *P*-value^*^ | 0.0580 | 0.0005 |  |  |  |
| Thoracic radiologist 4 | 0.958  (0.928-0.988) | 0.899  (0.857-0.940) | 0.928  (0.849-0.973) | 0.940  (0.874-0.978) | 0.818  (0.742-0.880) |
| *P*-value^*^ | 0.0089 | 0.0001 |  |  |  |
| Thoracic radiologist 5 | 0.959  (0.929-0.989) | 0.914  (0.874-0.953) | 0.940  (0.865-0.980) | 0.940  (0.874-0.978) | 0.856  (0.784-0.911) |
| *P*-value^*^ | 0.0092 | <0.0001 |  |  |  |
| Session 2 (Physician with DLAD assistance) | | | | | |
| Thoracic radiologist 1 | 0.980  (0.959-1.002) | 0.962  (0.937-0.987) | 0.964  (0.898-0.993) | 0.960  (0.901-0.989) | 0.932  (0.875-0.968) |
| *P*-value^†^ | 0.3123 | 0.1430 | 1.0000 | 0.1573 | 0.0833 |
| Thoracic radiologist 2 | 0.982  (0.961-1.002) | 0.946  (0.917-0.975) | 0.964  (0.898-0.993) | 0.990  (0.946-1.000) | 0.894  (0.829-0.941) |
| *P*-value^†^ | 0.3779 | 0.4845 | 0.3173 | 0.0833 | 0.6547 |
| Thoracic radiologist 3 | 0.982  (0.964-1.001) | 0.941  (0.906-0.976) | 0.988  (0.935-1.000) | 0.850  (0.765-0.914) | 0.917  (0.856-0.958) |
| *P*-value^†^ | 0.1486 | 0.0277 | 0.0832 | 0.1573 | 0.0253 |
| Thoracic radiologist 4 | 0.967  (0.940-0.995) | 0.927  (0.891-0.963) | 0.952  (0.881-0.987) | 0.940  (0.874-0.978) | 0.864  (0.793-0.917) |
| *P*-value^†^ | 0.1704 | 0.0227 | 0.3173 | 1.0000 | 0.0339 |
| Thoracic radiologist 5 | 0.974  (0.950-0.998) | 0.932  (0.898-0.965) | 0.964  (0.898-0.993) | 0.940  (0.874-0.978) | 0.886  (0.820-0.935) |
| *P*-value^†^ | 0.0991 | 0.0226 | 0.1573 | 1.0000 | 0.1797 |

Abbreviations: AUROC, area under the receiver operating characteristic curve; AUAFROC, area under the alternative free-response receiver operating characteristic curve; DLAD, deep-learning based automatic detection algorithm.

^*^Comparison of performance with DLAD

^†^Comparison of performance with session 1
